# *Daphnia magna* transcriptome by RNA-Seq across 12 environmental stressors

**DOI:** 10.1038/sdata.2016.30

**Published:** 2016-05-10

**Authors:** Luisa Orsini, Donald Gilbert, Ram Podicheti, Mieke Jansen, James B. Brown, Omid Shams Solari, Katina I. Spanier, John K. Colbourne, Douglas Rush, Ellen Decaestecker, Jana Asselman, Karel A.C. De Schamphelaere, Dieter Ebert, Christoph R. Haag, Jouni Kvist, Christian Laforsch, Adam Petrusek, Andrew P. Beckerman, Tom J. Little, Anurag Chaturvedi, Michael E. Pfrender, Luc De Meester, Mikko J. Frilander

**Affiliations:** 1 Environmental Genomics Group, School of Biosciences, University of Birmingham, Birmingham B15 2TT, UK; 2 Biology Department, Indiana University, 1001 E. Third Street, Bloomington, Indiana 47405, USA; 3 School of Informatics and Computing, Indiana University, 919 E. Tenth Street, Bloomington, Indiana 47408, USA; 4 Center for Genomics and Bioinformatics, Indiana University, School of Informatics and Computing, Indiana University, 1001 E. Third Street, 919 E. Tenth Street, Bloomington, Indiana 47408, USA; 5 Laboratory of Aquatic Ecology, Evolution and Conservation, University of Leuven, Ch. Deberiotstraat 32, Leuven 3000, Belgium; 6 Department of Genome Dynamics Lawrence Berkeley National Laboratory, University of California Berkeley, Berkeley, California 94720, USA; 7 Aquatic Biology, Interdisciplinary research Facility Life Sciences KU Leuven Campus Kortrijk, E. Sabbelaan 53, Kortrijk B-8500, Belgium; 8 Laboratory of Environmental Toxicology and Aquatic Ecology, GhEnToxLab,Ghent University, Ghent, Belgium; 9 Universität Basel, Zoologisches Institut, Vesalgasse 1, Basel 4051, Switzerland; 10 Centre d’Ecologie Fonctionnelle et Evolutive—CEFE UMR 5175, CNRS—Université de Montpellier—Université Paul-Valéry Montpellier—EPHE, campus CNRS, 1919, route de Mende, Montpellier, Cedex 5 34293, France; 11 Institute of Biotechnology, University of Helsinki, PO Box 56, Viikinkaari 9, 00014, Helsinki Finland; 12 Animal Ecology I and Bayreuth Center of Ecology and Environmental Research (BayCEER), University of Bayreuth, Bayreuth 95440, Germany; 13 Department of Ecology, Faculty of Science, Charles University in Prague, Viničná 7, Prague CZ-12844, Czech Republic; 14 Department of Animal and Plant Science, University of Sheffield Alfred Denny Building, Western Bank, Sheffield S10 2TN, UK; 15 Ashworth Laboratories, Institute of Evolutionary Biology, University of Edinburgh, Kings Buildings, Edinburgh EH9 3JT, UK; 16 Department of Biological Sciences and Environmental Change Initiative, Galvin Life Science Center, Notre Dame, Indiana 46556, USA

**Keywords:** RNA sequencing, Computational biology and bioinformatics

## Abstract

The full exploration of gene-environment interactions requires model organisms with well-characterized ecological interactions in their natural environment, manipulability in the laboratory and genomic tools. The waterflea *Daphnia magna* is an established ecological and toxicological model species, central to the food webs of freshwater lentic habitats and sentinel for water quality. Its tractability and cyclic parthenogenetic life-cycle are ideal to investigate links between genes and the environment. Capitalizing on this unique model system, the STRESSFLEA consortium generated a comprehensive RNA-Seq data set by exposing two inbred genotypes of *D. magna* and a recombinant cross of these genotypes to a range of environmental perturbations. Gene models were constructed from the transcriptome data and mapped onto the draft genome of *D. magna* using EvidentialGene. The transcriptome data generated here, together with the available draft genome sequence of *D. magna* and a high-density genetic map will be a key asset for future investigations in environmental genomics.

## Background & Summary

Illuminating the link between genes and environment is an exciting yet challenging goal. The full exploration of this link requires model organisms with well-characterized ecological interactions in nature, tractability in the laboratory and available genomic tools. The waterflea *Daphnia magna* Straus satisfies these requirements^1,2^. *D. magna* occurs in lakes and ponds in Europe, Africa, Asia and America^3,4^. It has a prominent ecological role in pelagic freshwater food webs, where it is the primary forage for many vertebrate and invertebrate predators^5–7^, an efficient grazer of algae^8^, including cyanobacteria^9^, a strong competitor for other zooplankters^10^ and in a constant evolutionary race with parasites^11^. Experimental tractability is high in *Daphnia* because of the short generation time, comparable to the genetic model species *Drosophila*. The small body size enables experimental approaches on large populations, and the cyclic parthenogenetic life cycle enables the parallel analysis of functional and fitness changes in the same genotype in multiple environmental conditions. Moreover, species of the genus *Daphnia* are renowned models in ecotoxicology and are widely used as indicators of water quality and environmental health^12–16^. They are also key models in evolutionary biology and the study of adaptive responses to environmental change^17–21^.

Capitalizing on this unique model system, the STRESSFLEA consortium, a research network funded by the ESF EUROCORES Programme EuroEEFG, generated a comprehensive RNA-Seq data set obtained from two natural genotypes, subsequently inbred in the laboratory, and a recombinant line of *D. magna*, obtained from the crossing of the two inbred genotypes, exposed to a suite of biotic and abiotic environmental perturbations. The two inbred genotypes were collected from two ecologically different habitats in the species distributional range. One of the inbred strains has been used to obtain the first draft genome of *D. magna* v2.4 (GenBank LRGB00000000).

Genome-wide transcription profiling was obtained from the three genotypes following environmental perturbations. The EvidentialGene method based on combined RNA-assembly and genome-based modelling of euGenes eukaryote genome informatics (http://eugenes.org/EvidentialGene/)^22^ was used to generate a public gene set for *D. magna* with as complete and accurate gene and transcript repertoire as possible. EvidentialGene uses evidence from public gene expression and protein datasets to annotate new genes. Briefly, for each gene, different models are tested and ranked based on quality scores and on deterministic evidence. Selecting the best representative model for a locus from among a large set of models is accomplished over two criteria: (1) gene evidence must pass a minimum threshold score, and (2) the combined score is maximal for all models overlapping the same coding sequence locations. Other criteria and tests are included and used for classification, such as orthology scores, CDS/UTR quality, and expression and intron evidence. The algorithm used for evidence scoring attempts to match expert choices, using base-level and gene model quality metrics. Determining a final gene set is an iterative process that involves evaluation and expert examination of problematic cases, modification of score weights, and reselection.

The data generated here combined with the *D. magna* draft reference genome and a genetic map available for this species^23^ will open a new era for environmental genomics. These genome and gene data sets are publicly available in the interactive *Daphnia* genome database at wFleaBase.org^24^. This database includes a genome map viewer with an option to display expression data (for example from this study) and genome annotation data from *Daphnia pulex* and related species, as well as search functions for queries at sequence, gene function, expression, orthology and annotation levels. The RNA-Seq data generated in this study will enable us to disentangle the relative contribution of genetic adaptation and phenotypic plasticity to adaptation in presence of both natural and anthropogenic stressors. Such investigations are possible because of the rich ecological data available for *Daphnia*, which is arguably the best studied model system in terms of phenotypic and genetic responses to ecological stressors^1,2^. In combination with the key assets of this model system for experimental work, the transcriptomic data deposited here will enable unprecedented advances in environmental, population and functional genomics.

## Methods

### Strains

Two inbred genotypes derived from natural strains, and a recombinant line derived from a cross of these two strains, were used to generate the transcriptome of *D. magna*. The two natural strains were collected from a system of ephemeral rock pools from the northern distributional range of the species (Xinb3, South west Finland 59.833183, 23.260387) and a fish-rearing pond in Southern Germany (Iinb1, Germany, 48.206375, 11.709727), respectively. The Xinb3 genotype was the result of three generations of selfing, and the Iinb1 strain was selfed for one generation, leading to a predicted 87.5 and 50% reduction in their original level of heterozygosity, respectively. The recombinant line is an F2 laboratory strain part of a mapping panel supporting research on the genetic basis of adaptive traits in *D. magna*^23,25^. The strains will hereafter be referred to as X- Xinb3, I- Iinb1, and XI-recombinant line.

### Environmental perturbations and experimental design

Genome-wide transcription profiles were obtained from the three strains following environmental perturbation by a suite of environmental challenges. Exposures to environmental perturbations on the two inbred strains were conducted at the University of Leuven, Belgium. The sequencing for this experiment was performed at the Finnish Institute of Molecular Medicine (FIMM, Technology Centre, Sequencing unit) at the University of Helsinki. Exposures of the recombinant line were completed at the University of Notre Dame, IN, USA. The sequencing data from this experiment were obtained at the JP Sulzberger Columbia Genome Center (https://systemsbiology.columbia.edu/genome-center). All exposures to environmental perturbations were conducted using the protocol described below. All three genotypes were maintained in the laboratory for several generations after selfing (X and I) or crossing (XI) to reduce interference from maternal effect prior to the exposures to environmental perturbations.

#### Inbred genotypes (X and I)

**Figure 1:**
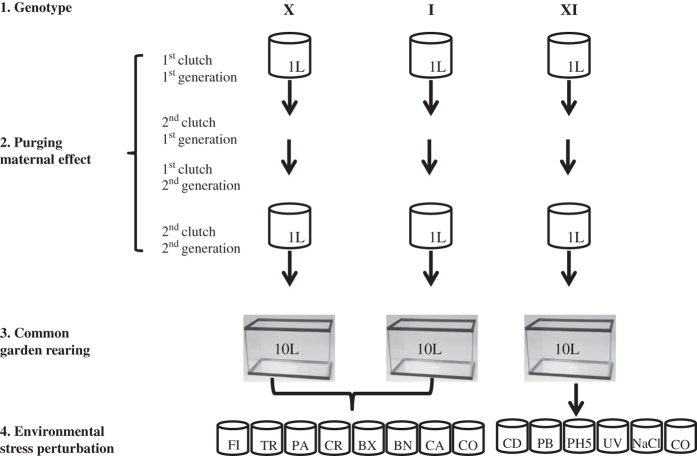
Workflow of environmental perturbations. Two natural genotypes of *D. magna* collected from Finland (Xinb3-X) and Germany (Iinb1-I) and a recombinant line (XI) obtained from the cross of the two natural genotypes were used in experimental perturbations. The three genotypes were synchronized for two generations. The second clutch of the second generation was exposed to environmental perturbations. The environmental perturbations for the natural genotypes were as follows: **FI**: Vertebrate predation mimicked by fish kairomones released by 19 sticklebacks in 100 l water; **TR**: Invertebrate predation mimicked by kairomones released by 1 adult *Triops* in 2 l water; **PA**: exposure to parasite spores by the common parasite *Pasteuria ramosa*—40,000 sporesml^−1^; **CR**: crowding exposure conditions are of 100 individuals/250 ml; **BX** Toxic Cyanobacteria—strain MT50; **BN** Non-toxic Cyanobacteria—strain CCAP 1450/1; **CA**: exposure to the pesticide Carbaryl—8 μgl^−1^; **CO**: control. The environmental perturbations for the recombinant line were as follows: **CD:** Cadmium-6 μgl^−1^; **PB**: Lead-278 μgl^−1^; **pH 5.5**; **UV** light; **NaCl**- 5 gl^−1^; **CO** Control.

For the exposure to environmental perturbations, the genotypes were grown in climate chambers with a fixed long day photoperiod (16 h light/8 h dark) at 20 °C. The first generation was cultured at a density of 10 individuals/l, and increased to 50 individuals/l in the second generation to enable the harvesting of enough offspring for the environmental perturbation exposure. Animals were harvested and exposed in ADaM medium (Aachener Daphnien Medium:^26^). The medium was renewed every second day in the harvesting phase and the daphnids were fed daily with 150,000 cells *Scenedesmus obliquus*/ml. The diet changed to a 2:1*S. obliquus*:*Cryptomonas* sp. mix from the second generation onwards to provide the animals with optimal food quality. When multiple genotypes were used in the same experimental set up, they were synchronized for at least two generations prior to the actual exposures. The second clutch of the second generation was used for exposures to environmental perturbations. Five-day old juveniles at a density of 100 juveniles/l were exposed for 4 h to the different environmental challenges (Fig. 1). Prior to separating the juveniles for the actual exposures, they were grown in groups of 1,000 in 10 l aquaria for four days. The aquaria were fed daily 150,000 cells per ml in a 2:1 *S. obliquus*: *Cryptomonas* sp. Half of the medium was replaced every second day. The animals were not supplied with food during the perturbation exposures to reduce contamination from algae in the sequencing phase. Seven environmental perturbations were imposed on the inbred strains. These consisted of six biotic and one abiotic stressor. The biotic stressors were: kairomone signalling of vertebrate and invertebrate predation, exposure to *Pasteuria ramosa* parasite spores, crowding, and grazing on toxic and non-toxic cyanobacteria; the abiotic stressor was the pesticide Carbaryl (1-napthyl methylcarbamate, Sigma-Aldrich, Germany) (Fig. 1). To mimic fish predation, *Daphnia* were exposed to kairomones-enriched medium obtained from growing 19 sticklebacks in 100 l of water. This medium was obtained from aquaria in which fish was reared. Medium in the fish aquaria was refreshed daily, and kairomone-loaded medium was prepared by filtering the medium over a 0.2 μm filter. This kairomone-loaded medium was added to the *Daphnia* cultures to constitute 10% of the total volume. Similarly, invertebrate predation was mimicked by exposing *Daphnia* to kairomones-enriched medium obtained from growing an adult tadpole shrimp *Triops* in 2 l of water. This medium was obtained by filtering the kairomone-loaded medium on a 0.2 μm filter. Similarly to the fish kairomone experiment, the filtered medium was added to the *Daphnia* cultures to constitute 10% of the total volume. Experimental animals in the parasite treatment were exposed to a solution containing 40,000 spores/ml of *P. ramosa*, a parasite known to have strong fitness consequences in *Daphnia*^11^. Crowding stress was imposed by increasing the number of experimental animals per volume of medium: 100 individuals in 250 ml of medium as compared to 100 individuals in 1 l. Perturbation from cyanobacteria was obtained by feeding *Daphnia* with a toxic strain of *Microcystis aeruginosa* (Cyanobacteria, strain MT50) and a non-toxic strain of *Microcystis aeruginosa* (strain CCAP 1450/1)^9^. The experimental animals were exposed to the pesticide Carbaryl in a concentration of 8 μgl^−1^, known to cause appreciable sublethal stress and increased mortality^27^. The exposures of the inbred strains were completed over two days. For each day a control (no stress) was run in parallel to the environmental perturbations. We performed five biological replicates for each treatment, including controls. Each consisted of ca. 80 sub-adult animals.

#### Recombinant genotype (XI)

The recombinant line was maintained as described above for the parental genotypes with the exception that recombinant *Daphnia* were maintained in 1 l containers throughout the rearing phases and for the experimental phase, third generation individuals at eight days old were exposed to five abiotic perturbations linked to anthropogenic disturbance. These exposures included: cadmium (Cd), lead (Pb), low pH (5.5), UV light, and sodium chloride (NaCl) (Fig. 1). The experimental treatments included a single control of individuals placed in fresh media without algae for a 24 h period. All treatments and the control included three biological replicates. The metal exposures were maintained for 24 h at concentrations of 6 μgl^−1^ and 278 μgl^−1^ for Cd and Pb, respectively. *Daphnia* were also exposed to pH 5.5 and media supplemented with 5 g/l NaCl for 24 h. UV light treatments were conducted in 250 ml beakers containing 50 ml of media. Beakers were placed 10.5 cm below 30 W, 36-inch Reptisun 5.0 UVB fluorescent light bulbs for 4 h (Zoo Med Laboratories Inc., San Luis Opispo, CA, USA)^20^. Exposure to UV light was restricted to 4 h to avoid high mortality observed at 24 h. All recombinant line exposures were conducted at 18 °C and RNA collection was timed to occur at the same time period to minimize circadian variation in expression patterns among treatments.

### RNA isolation

#### Inbred genotypes (X and I)

Five biological replicates for each genotype were perturbed with the environmental conditions explained above and RNA-Seq generated from three of the five biological replicates. Having a larger set of exposed biological replicates per genotypes allowed us to choose the three replicates with the highest RNA quality. Total RNA was extracted from pools of ca. 80 juveniles from each genotype and replica by homogenization in the presence of Trizol reagent followed by acidic phenol extraction as described in (ref. 28) and ethanol precipitation. Quality of the isolated RNA was confirmed with Bioanalyzer (Agilent Technologies, Santa Clara, CA, USA) and only samples showing no RNA degradation were used in subsequent steps. Sequencing was performed on 49 samples: 3 replicates x 2 genotypes x 8 conditions (2 controls were run for genotype X, making the total number of run samples 49, as the environmental exposures were spread over two days).

#### Recombinant genotype (XI)

Total RNA was extracted from pools of ca. 50 individuals from each replicate (18 samples: 3 replicates x 6 conditions including control) by homogenization in Trizol reagent and isolating RNA using a Qiagen RNeasy column (Qiagen, Valencia, CA, USA) with on column DNase treatment. RNA quality was assessed as above.

### Construction of RNA-seq libraries

The experimental procedure from library construction to sequencing and downstream analysis was identical for the three genotypes and was as follows.

Library construction was performed on three biological replicates. 1–3 μg of total-RNA was used for isolating poly-A RNA (Dynabeads mRNA purification kit, Ambion, Life Technologies, AS, Oslo, Norway). The poly-A RNA was reverse transcribed to ds-cDNA (SuperScript Double-Stranded cDNA Synthesis Kit, Life Technologies, Carlsbad, CA, USA). Random hexamers (New England BioLabs, Ipswich, MA, USA) were used for priming the first strand synthesis reaction and SPRI beads (Agencourt AMPure XP, Beckman Coulter, Brea, CA, USA) for purification of cDNA. Illumina compatible Nextera Technology (Illumina, Inc., San Diego, CA, USA) was used for preparation of RNA-seq libraries. 60 ng of ds-cDNA was fragmented and tagged using *in vitro* cut-and-paste transposition. The fragmented cDNA was purified with SPRI beads. In order to add the Illumina specific bridge-PCR compatible sites and enrich the library, limited-cycle PCR (5 cycles) was done according to instructions of Nextera system with minor modifications. For bar coded libraries, 50X Nextera Adaptor 2 was replaced with a bar coded Illumina-compatible adaptor from the Nextera Bar Codes kit in PCR setup. SPRI beads were used for purification of the PCR-products and the library QC was evaluated by Agilent Bioanalyzer. Libraries were size selected (350–700 bp) in 2% agarose gel, purified with QIAQuick Gel Extraction kit (Qiagen, Valencia, CA, USA) and the library QC was evaluated by Agilent Bioanalyzer.

### RNA-seq library sequencing

C-Bot (TruSeq PE Cluster Kit v3, Illumina, San Diego, CA, USA) was used for cluster generation and Illumina HiSeq2000 platform (TruSeq SBS Kit v3 reagent kit) for paired-end sequencing with 101 bp read length. Sequence data for the inbred genotypes were generated by the FIMM sequencing unit at the University of Helsinki, Finland whereas data from the recombinant genotype were generated by the JP Sulzberger Columbia Genome Center (New York, NY, USA).

### RNA-Seq quality check

Read sequences were subjected to adapter trimming and quality filtering using Trimmomatic ver.0.33 (ref. 29). RNA-Seq reads were checked for foreign RNA contamination. Human and mouse contaminant sequences were screened and removed by mapping *D. magna* reads onto ncbigno2014-human.rna and ncbigno2014-mouse.rna using bowtie2 ver.2.1.0 (ref. 30). Finally, 80% of the reads for the inbred genotypes and 99% of the reads for the recombinant genotype were retained (Q>20). Contaminant screening is essential for transcriptome and genome projects; in this study contaminants of 100% RNA identity to mouse, human, and various bacteria genes were found in all source sets, even though not in all replicates. Care should also be taken to avoid false positive contaminant flags, as putative horizontal gene transfer (HGT); one such case was identified in the current dataset. The cleaned reads were mapped onto the reference transcriptome of *D. magna* obtained from *de novo* assembly of RNA-Seq data. These data consisted mostly of the Xinb3 inbred genotype data, but also included a subset of data from the Iinb1 genotype and RNA-Seq available in public databases for *D. magna* at the time of the analysis (mostly^31^). This reference transcriptome includes only primary transcripts. The mapping of reads from the three genotypes was conducted using Bowtie2 ver.2.1.0 (ref. 30) allowing a maximum edit distance of 3 per read. 74% of the reads mapped on the reference transcriptome and 82% of those mapped to a unique location. The remaining reads mapped to multiple locations suggesting that those are alternative transcripts or incomplete genes that cannot be accurately mapped. These reads will be the object of further investigations.

As an additional assessment of sequence quality, we counted base positions in which more than two allelic variants were present, hence departing from the expectation of a maximum of two alleles at a given position for a diploid organism. For this analysis reads from all treatments for each genotype were pooled and mapped against a reference sequence set of single copy genes from the *D. magna* consensus transcriptome. The mapping process was performed using bowtie2 ver. 2.1.0 30 reporting up to a maximum of 20 valid alignments per read (-k 20); from this pool, alignments with least edit distance were selected as best hits for a specific read. Allelic variants as compared to the reference consensus sequence were identified using samtools mpileup command (samtools ver. 0.1.19, 45), and a custom parser written in perl. The minimum base quality score required for a variation to be considered was q=20 where q is the threshold measured. Variant calls with frequencies below 1% representing typical Illumina sequencing errors^32^ were excluded. The variant positions with 2, 3 or 4 allelic variants were counted.

### Transcriptome and gene set construction

#### Transcriptome assembly of RNA

We used EvidentialGene methods from the euGenes.org^22^ project to assemble RNA-seq, as well as annotate and validate transcripts per strain. After assembling transcripts per strain, we constructed a complete gene set across strains incorporating chromosome assembly data available for *D. magna* (draft genome assembly 2.4, GenBank LRGB00000000). Paired end RNA-Seq reads, totalling 7.2 billion reads from the current project and 2 billion reads from published data at the time of the analysis^31^, were assembled *de-novo* with several RNA assemblers, using multiple options for kmer fragmenting, insert sizes, read coverage, digital normalization, and quality and abundance filtering. *De-novo* RNA assemblers used include Velvet/Oases^33,34^ [v1.2.03/o0.2.06], SOAPDenovo-Trans^35^ [v2011.12.22] using multi-kmer shredding options from 23 to 95 bp, and Trinity^36^ [v2012.03.17] (with fixed kmer option). Accessory methods used for RNA data processing include GMAP/GSNAP and Bowtie for read and transcript mapping to genome assembly, diginorm of khmer package, and sequence artefact filtering. Additional transcripts were assembled with genome-mapping assistance, using PASA^37^ [v2.2011], Cufflinks^38^ [v1.0.3 and v0.8], and EvidentialGene. EvidentialGene *tr2aacds* software pipeline (http://eugenes.org/EvidentialGene/trassembly.html) was used to process the resulting assemblies obtained from coding sequences. The assemblies were then translated to proteins, scored for gene evidence including CDS/UTR quality and homology, and reduced to a biologically informative transcriptome of primary and alternate transcripts. We submitted to NCBI only the primary transcripts; alternate transcripts are available at wFleaBase.org.

#### Gene set construction

Gene models were also predicted on the draft *D. magna* genome assembly with genome-modelling methods, using AUGUSTUS^39^, and were incorporated in this public gene set version evg7f9b. Accessory gene set annotation, validation and processing methods included NCBI BLAST suite, exonerate (protein alignment), lastz (sequence alignment), GMAP (gene mapper), CD-Hit (sequence clustering), MUMmer (sequence alignment), MCL (markov clustering), Muscle (sequence alignment), RepeatMasker (repeat and transposon finding), rnaexpress, samtools (rna), SNAP (gene modeller), Splign (alignment), and several database extracts of arthropod and eukaryote genes, proteins and other sequences. A set of primary and alternate transcripts per locus was determined with CDS-overlap discrimination and weighted sum of the several gene evidence scores per transcript model. In hybrid gene set constructions, such as the one presented here, errors occur from both genome map modelling and mRNA assembly, and discrepancies between methods need to be resolved from available gene evidence. The algorithm used for this gene set construction was Evidential Gene and includes three stages:

1. **Stage 1.** Transcript assemblies of mRNA-seq are performed with several *de-novo* assemblers and parameters, followed by EvidentialGene tr2aacds redundancy removal for each assembly set.
2. **Stage 2.** Locus/alternate gene classification is performed from assembly sets obtained in stage 1 to produce non-redundant gene assemblies for each strain using several attributes: transcript alignment classification (tr2aacds), genome-map location and consensus map loci, consensus protein homology and quality, and cross-strain transcript consensus (MCL clustering of transcript alignments^40^).
3. **Stage 3.** A candidate locus/alternate gene set for the species is produced from the non-redundant strain sets, using several gene consensus measures across strains, expert curation and computational reclassification. Cases of alternate/paralog discrimination and mis-mapping are investigated in this step using consensus of gene structure among strains, protein orthology analyses, and consensus location on *D. magna* and the sister species *D. pulex* chromosome assemblies.

Stage1 produced separate RNA assemblies for the three genotypes, amounting to 16.5 M transcripts for X, 9.5 M for I, and 3.7 M for XI, plus a 4th genome-assisted de-novo assembly of 1 M transcripts from weak expressed loci (X genotype). Stage 2 produced 1.0 M non-redundant mRNA transcripts ranging from 35,000 to 270,000 transcripts per set across 7 gene sets obtained from strain and genome-based inferences. The gene set obtained in this second stage is derived from 30 million mRNA assemblies obtained in stage 1. Stage 3 involved cross-strain consensus locus determination, including paralog/alternate discrimination, iterative reclassification and refinements, reducing the total set to 29,128 loci and primary transcripts, with 84,882 alternative transcripts found among 17,473 of those loci.

Gene homology evidence for the gene construction pipeline includes 300,000 proteins from 10 species: the waterfleas *Daphnia magna* and *Daphnia pulex* (version 2010, wFleaBase.org), the tiger shrimp *Penaeus monodon* (2013 EvidentialGene), the flour beetle *Tribolium castaneum* (2014 NCBI), the beetle *Pogonus chalceus* (2013 EvidentialGene), the honeybee *Apis mellifera* (2014 NCBI), the wasp *Nasonia vitripennis* (2010 EvidentialGene), the fruitfly *Drosophila melanogaster* (rel5.30 2012), the fish *Maylandia zebra* (NCBI 2014) and humans (UniProt 2011). Orthology and paralogy criteria were assigned using all versus all reciprocal blastp of these species, followed by OrthoMCL^41^ alignment clustering of genes (Dmag analysis version arp7bor5 in wFleaBase.org). Gene names were assigned to our models on the basis of homology scores to UniProt proteins. The consensus gene family names were obtained from OrthoMCL orthology analyses, in accordance with UniProt protein naming guidelines^42^.

The basic approach employed by EvidentialGene is similar to other eukaryote genome annotation methods, including NCBI Eukaryote genome annotation pipeline^43^ (http://www.ncbi.nlm.nih.gov/genome/annotation_euk/process/), ENSEMBL genome annotation pipeline (http://www.ensembl.org/info/genome/genebuild/genome_annotation.html), TIGR and Broad genome annotation software^44^, and MAKER^45^. It differs from these other approaches for its deterministic evidence scoring, detailed per gene annotations, and single-best model/locus approach. A notable divergence from these other methods is the use of hybrid mRNA-assembly and genome modelling which increases the accuracy and completeness of the gene sets generated.

### Assessing the gene set completeness

Orthology completeness, presence and full length of orthology genes were assessed with OrthoMCL in several steps of the gene set construction and in particular during stage 3 (Table 1). For an independent quantitative assessment of orthology completeness we used BUSCO (Benchmarking Universal Single-Copy Orthologs, v1.1, http://busco.ezlab.org/^46^), a recognized benchmark approach for single copy orthologs providing an assessment of orthologs conserved among species. Deviations from completeness are commonly interpreted as technical or, less frequently, biological deviations from the expected gene complement. We compared the gene models of *D. magna* (dmagset7finloc9b.mRNA gene set) with the BUSCO arthropod profiles. In addition, we compared our gene model with the one of four other arthropod species including *Daphnia pulex*, *Apis mellifera, Tribolium castaneum* and *Drosophila melanogaster*. Our analysis includes also multiple genes sets from the same species. Different genes sets are identified with year and source: 1) **Dma_14EV** described here using EvidentialGene methods, 2) **Dma_11G** obtained from genome-modelled *D. magna* genes from 2011 (this gene set will be described in a separate paper presenting the first draft genome of *D. magna*), 3) **Dpu_10EG** and 4) **Dpu_07G** available for *D. pulex*; 5) **Ame_14EV** obtained from *Apis mellifera* RNA-seq publicly available using EvidentialGene methods; 6) **Ame_12G** apis45: OGS v3.2 genome genes; 7) **Tca_14EV** obtained from *Tribolium castaneum* RNA-seq publicly available using EvidentialGene methods; 8) other Ame and Tca publicly available gene sets; 9) **Fly13** and **Fly04** generated in 2013 and 2004 for *Drosophila melanogaster*. An in depth analysis of the different gene sets and discussion of reliability of validation methods will be presented elsewhere.

## Data Records

*Daphnia magna* transcriptome and related data are published under the International Nucleotide Sequence Database Collaboration BioProject PRJNA284518 (http://www.ncbi.nlm.nih.gov/bioproject/?term=PRJNA284518). The *D. magna* consensus transcriptome for each of the three genotypes studied here and the raw data for each library obtained from different environmental perturbations are deposited in GenBank (Data Citation 1, metadata in Supplementary Table 2). RNA-seq read and transcript assemblies of RNA-Seq data can be found at this entry. Transcript assemblies generated separately for the two inbred strains are also available at GenBank (Daphnia magna Xinb3, Data Citation 2; Daphnia magna Inb1, Data Citation 3). The X assembly contains 42,990 loci with 253,834 transcripts.

The I assembly contains 36,935 loci with 271,331 transcripts. The X and I annotated assemblies contain coding-sequence validated for primary and alternate transcripts from stage 2 in the EvidentialGene pipeline above (Table 1, Dapma6tx and Dapma6ti clone sets), with loci determined by shared exons. Links to public gene set IDs are included with each transcript assembly. The complete hybrid mRNA-assembly and genome-modelled *D. magna* gene set, and draft genome assembly, in standard sequence and GFF annotation data formats, is publicly available at http://wfleabase.org/genome/Daphnia_magna/openaccess/genes/. The *D. magna* gene proteins are also available at UniProt http://www.uniprot.org/uniprot/?query=taxonomy:35525.

## Technical Validation

### Metrics of RNA—seq data

A total of 7.2 billion reads were generated, with an average of 107.5 million reads per sample (s.d. 22 million read pairs). The number of reads was 3.5billion (1.75 billion read pairs) for the X, 2.8 billion (1.4 billion read pairs) for I, and 0.8 billion (0.4 billion read pairs) for XI. Of the total number of reads, 77% for X, 81% for I and 77% for XI had quality scores above 30 (analysed with FastQC software^47^, Table 2). In Table 3 (available online only) we show a detailed analysis of the RNA-Seq data per sample including raw data read pairs before and after trimming quality was applied, as well as insert size. Approximately 70% of the reads retained their full length of 101 bases after trimming (Table 3) (available online only). Insert size for each paired-read library was estimated by mapping a random subsample of 1,000,000 reads per sample to the mitochondrial genome sequence on the reference draft genome (reference genome ver.2.4). The size of the insert for each concordantly mapped read pair was estimated and the average drawn over all such read pairs. The insert size for each sample is shown in Table 3 (available online only).

**Figure 2:**
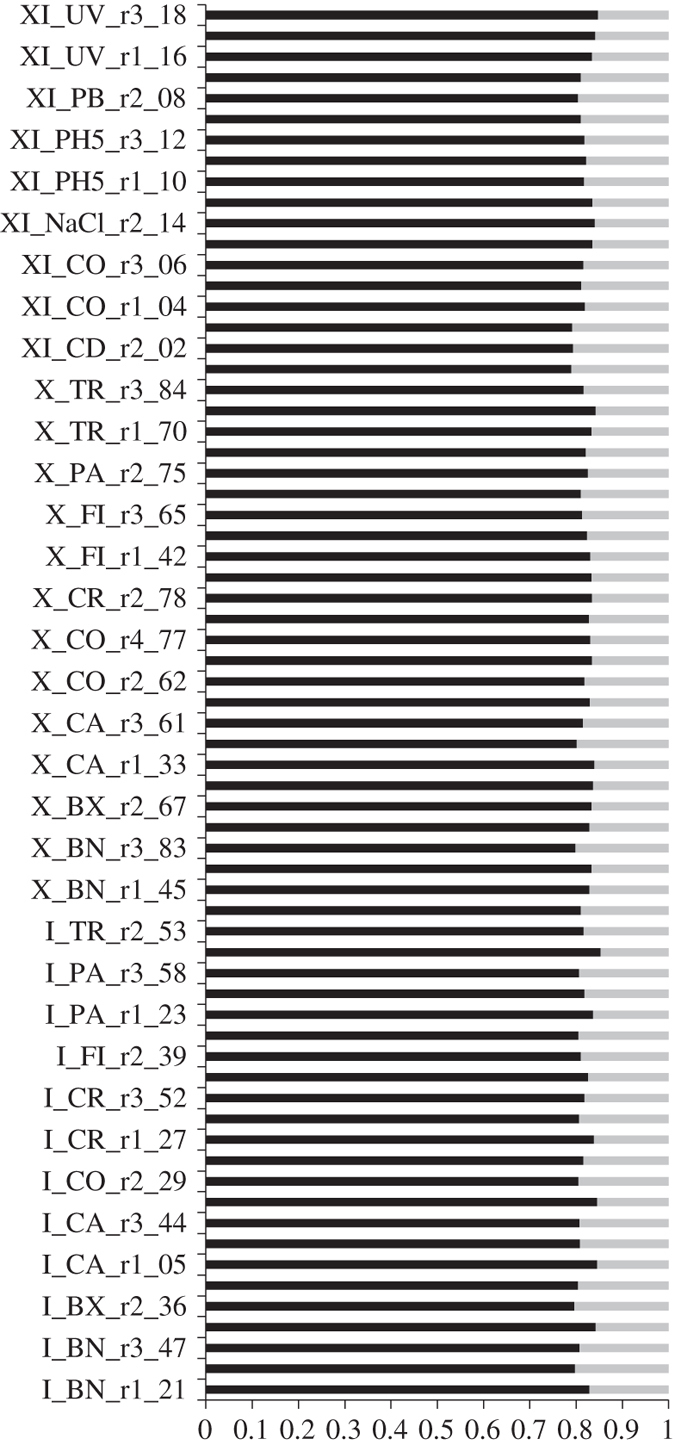
Percentage of mapped read pairs. Percentage of read pairs per samples mapping to unique (black bars) or to multiple locations (grey bars) in the reference transcriptome of *D. magna.*

When using primary transcripts only, the number of reads mapping onto the transcriptome ranged between 61 and 78% (Fig. 2, Table 4 (available online only)). This percentage reached 98% of all reads when primary and alternate transcripts were used (Table 5). If the same read mapped multiple times onto the same transcript, it was counted only once for that transcript. Multiple mapped reads can be alternate transcripts of the same gene or the result of incomplete mapping likely caused by partial sequence of a transcript. The reads mapping to multiple locations will be object of future studies and hence are not discussed further. The read counts per gene ID are shown in Supplementary Table 1.

The total number of transcripts retained in this study after trimming and quality checks mapped onto 29,128 genes identified with the EvidentialGene model described above. The distribution of read pairs per gene is summarized in Supplementary Table 1. Between 26,508 and 28,187 transcripts were retrieved across the three genotypes (Table 6). The coverage in bp was highest for the X genotype with 5,282.66 and lowest for the XI genotype with 1,952.93 bp (Table 6). The difference in transcript-read map rates indicated in Tables 4 and 5 results from two main factors: (a) alternate transcripts account for 15% of the difference (all versus primary in Table 5) and (b) roughly a 10% difference in mapping of primary transcripts can be observed when different methods are adopted. For example GSNAP trims read ends to facilitate alignment to reference similarly to transcript assembly methods that trim and shred reads, whereas other methods like Bowtie do not trim ends.

### Allelic variants

After removing base positions with frequency lower than 1% which can be explained as sequencing errors^32^, we identified allelic variants with 2 to 4 alleles as compared to the reference set of single copy genes. The large majority of variants had one or two alleles as expected for a diploid organism (Table 7), confirming the high quality of our sequences. A small fraction of variants had 3 and 4 alleles. When a cut-off value of 5% on allelic variant calls was applied these variants were further reduced in number. From visual inspection of the alignment we assessed that these variants interested a very small fraction of the transcriptome.

### Reproducibility of biological replicates

**Figure 3:**
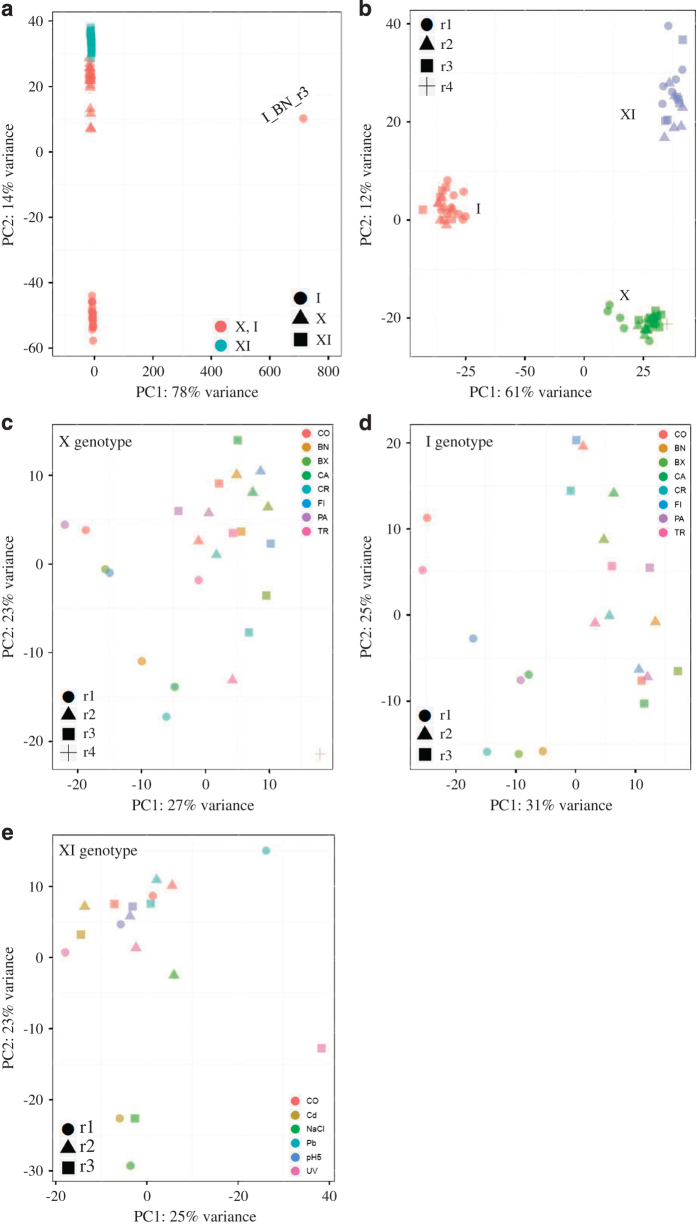
PCA plots. (**a**) PCA plots including all data, three genotypes (X, I, XI) and their biological replicates; genotypes X and I are in green, whereas genotype XI is in orange. The outlier treatment is the non-toxic cyanobacteria treatment on the I genotype (I_BN_r3 in panel **a**); (**b**) PCA plot including the three genotypes and their biological replicates except for the outlier HS_BN_Ir3; (**c**) PCA plot for the genotype X and its biological replicates; (**d**) PCA plot for the genotype I and its biological replicates; (**e**) PCA plot for the XI genotype and its replicates. Treatments short names are as in Fig. 1; they are depicted with different colours within each panel. The replicates are identified by different symbols.

A Principal Component Analysis on trimmed transcripts was used to assess the quality of the RNA-Seq data in terms of reproducibility across the biological replicates. The PCA plot inclusive of all data identified the sample I_BN_r3 as an outlier (Fig. 3a). This sample was removed from downstream analysis as it obscured any signal from both the genotype and the treatment. The PCA plots excluding this outlier showed a clear aggregation of replicates per genotype (Fig. 3b). PCA plots produced separately per natural genotype showed a roughly random distribution of the read counts along the two principal components (Fig. 3c,d) with a tendency of the first replica (r1) to cluster apart from the other two replicates. This may be the effect of slightly earlier developmental stage in r1 as compared to the other two replicas. In the PCA plot of the recombinant line (Fig. 3e), three treatments cluster separately from the others contributing more than 20% to the overall variance along both axes. These are the treatments with exposures of 24 h.

### Gene models validation

We generated a public gene catalogue for *D. magna* version evg7f9b1, for release to the scientific community. This hybrid gene set produced from both mRNA and genome gene models is available at wFleaBase.org with components available in International Nucleotide Sequence Database (INSDC).

Of the total 29,128 gene loci identified in *D. magna*, 26,825 (92%) genes were assembled from mRNA, and 2,296 (8%) were genome-modelled. 22,059 (76%) of the total recovered genes were complete proteins, and 7,068 (24%) partial proteins. All of these loci are supported by mRNA-Seq and/or protein homology evidence; 65% (18,962) of these genes map completely onto the *D. magna* draft genome assembly 2.4, and 99% (28,127) contains RNA-Seq reads unique to a specific locus. 76% of the total gene loci identified in *D. magna* show homology to other species (blastp e<=1e-5 to proteins or conserved domains) and 18% (5,170) show homology only to other *Daphnia* species. Finally, 40% of the recovered genes were orthologs to other species using orthology criteria of OrthoMCL, and 16% were paralogs of orthologs. 44% (12,826) of the total set of identified gene loci do not cluster with other species genes. This proportion can be considered unique or evolved in *D. magna*, although many genes derive homology from other species. The high number of *Daphnia* evolved genes is not unexpected considering the large number of eco-responsive genes identified in the related congener *D. pulex*^48^ and the fact that *Daphnia* species are among the first crustaceans with a draft genome sequence obtained from exposures to ecological stressors. We used the draft genome assembly of *D. magna* v 2.4 as part of the gene construction and validation process. Of this finished gene set, 65% (18,962) map properly onto the assembly with a coverage >=80%; 35% (10,189) of the genes mapped with low quality scores; 12% (3,389) remained un-mapped, 12% (3,386) partially-mapped, and 12% (3,414) showed split-mapping. These mapped genes include hundreds of trans-spliced and anti-sense loci where mRNA/protein and introns have reversed orientation. Finally, 14% of the genes that could be mapped were single-exon loci. Some of the conflicts among the physical map in the genome assembly v2.4, partially mapped genes and other complexity are artifactual results of draft genome missassemblies. Other of these complexities are located on well assembled portions, including the anti-sense transcription, and appear as true biological complexities. An instance of putative horizontal gene transfer, bacteria to *Daphnia*, was uncovered during contaminant screening. This has been reported in *Daphnia pulex*^49^ as a kairomone-stress responsive horizontal gene transfer (HGT) gene, and appears to exist in the draft genome of *D. magna*, *D. pulex,* and in *D. galeata* (personal observation DG). An automated contaminant screening flagged this as a contaminant, but further examination of evidence indicates probable *Daphnia* genomic source, with a potential ecological relevance of this gene to *Daphnia* species.

### Assessing the transcriptome annotation completeness

**Figure 4:**
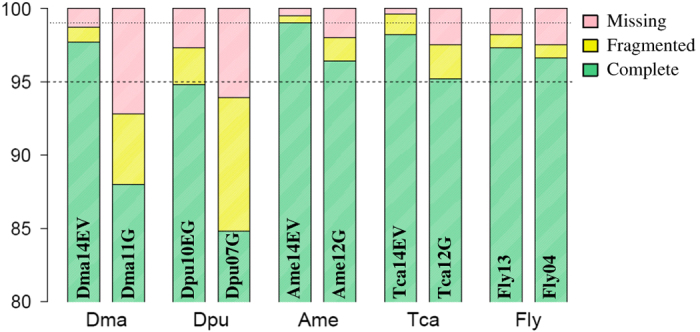
BUSCO analysis. Stacked bar plots showing proportions of gene sets in quality categories for *D. magna* and 4 other arthropod species.. Two gene sets are represented per species, as described in methods, to show effects of construction methods on quality. The categories of genes are: i) complete single copy BUSCO: genes which match a single gene in the BUSCO reference group; ii) fragmented BUSCOs: genes only partially recovered for which the gene length exceeds the alignment length cut-off; iii) missing BUSCO: not recovered genes. Abbreviation for species names are as follows: Dma=*Daphnia magna*; Dpu=*Daphnia pulex*; Ame=*Apis mellifera*; Tca=*Tribolium castaneum*; Fly=*Drosophila melanogaster*. The gene sets sources used for the 4 arthropod species are as follows: 1) **Dma_14EV** dapmagevg14:, Evigene mRNA+genome, 2014.08; 2) **Dma_11G** dapmag11: Evigene genome genes, 2011; 3) **Dpu_10EG** dapplx10evg: Evigene genome genes, 2010; 4) **Dpu_07G** dapplxjgiv11: genome genes, 2007, doi: 10.1126/science.1197761; 5) **Ame_14EV** apisevg14: Evigene mRNA assembly 2014.06; 6) **Ame_12G** apis45: OGS v3.2 genome genes, 2012, doi: 10.1186/1471-2164-15-86; 7) **Tca_14EV** tribcas4evg2: Evigene mRNA assembly, 2014.12; 8) **Tca_12G** tribcas4aug: AUGUSTUS genome genes, tcas4.0, 2014; 9) **Fly_13** drosmel548n: Flybase release 5.48, 2013; 10) **Fly_04** drosmelr4: Flybase release 4.0, 2004.

Evidence of high quality and completeness of the *D. magna* genes was provided by both OrthoMCL and the BUSCO analyses (Table 8 and Fig. 4). According to the OrthoMCL assessment, the current *D. magna* genes are as or more complete than related arthropods gene sets, with few orthologs missing, a higher number of complete genes, and a lower number of fragment outliers detected (Table 8). In the BUSCO analysis *D. magna* gene set showed the lowest proportion of missing and fragmented single copy orthologs as compared to the other four arthropod but for two other sets: Ame14evg and Tca14evg (Fig. 4). Notably, the species showing the most complete gene sets in our analysis were the ones in which the EvidentialGene methods was applied. A complete analysis of this method’s performance versus other methods is beyond the scope of the present paper and will be discussed elsewhere.

The STRESSFLEA consortium was a collaborative network of 10 Universities, including 7 European and 2 North American Universities. The effort of this consortium allowed us to produce a comprehensive transcriptome data set and a frozen gene catalogue for the premier model system *D. magna*. This effort paves the way to powerful discoveries in environmental and functional genomics elevating *D. magna* to the rank of genomics empowered ecological model species.

## Additional Information

**How to cite this article:** Orsini, L. *et al.* *Daphnia magna* transcriptome by RNA-Seq across 12 environmental stressors. *Sci. Data* 3:160030 doi: 10.1038/sdata.2016.30 (2016).

## Supplementary Material

Supplementary Table 1

Supplementary Table 2

## Figures and Tables

**Table 1:** *Daphnia magna* gene set generation

**Input_Tr**	**NR_out**	**Name**	**Source**
Stage 1			
3,751,425	140192	dmagset36m	Labbe *et al.* 2012May (Dapma6rm, daphmag3, dmag2vel, tag41 id patt)
16,454,489	256607	dmagset56tx	X assembly, 2014Jun-2013Aug (Dapma6tx, hsX, ndX, vel4x id patt)
9,469,773	272398	dmagset56ri	I assembly, 2014May (Dapma6ti,hsI,vel4i id patt)
1,000,000	64487	dmagset56ru	Assembly from X weakly expressed genes, 1st pass unassembled reads 2014Jun (Dapma6rx, xun, nun id patt)
Stage 2			
34530		dmagset1m8	Genome predicted 2011 (m8AUG id patt)
140192		dmagset36m	Labbe *et al.* rna 2012 May (Dapma6rm, daphmag3, dmag2vel, tag41 id patt)
256607		dmagset56tx	X assembly, 2014Jun-2013Aug (Dapma6tx, hsX, ndX, vel4x id patt)
272398		dmagset56ri	I assembly, 2014 May (Dapma6ti,hsI,vel4i ids) of 9469773 input trasm
64487		dmagset56ru	Assembly from X weakly expressed genes, 1st pass unassembled reads 2014 Jun (Dapma6rx, xun, nun ids)
120122		dmagset4pub1208	Present study rna data 2012 Aug, X mostly, used to fill in missed loci
182909		dmagset5xpub1401	Pre-release 2014Jan, used to fill in missed loci, from 2013–2010 transcripts
Stage 3			
**Name**	**nLoci**	**Notes**	
pubset1	97140	evg7vose-tr2aacds, input of 4 separately assembled and reduced RNA-seq sets (3-clones) and genome-predict set, no-omcl 04Jul2014. Sets 4 (1208) and 5 (1401) were not pubset1 inputs.	
pubset2	44762	no-omcl 24Jul2014; cross-clone consensus classification (MCG loci/alts common across clone sets)	
pubset3	28363	arp7aor1 orthology set, 30Jul2014	
pubset4	27239	no-omcl 14Aug2014; intron-miss loci, paralog/alt reclass	
pubset5	27218	no-omcl 19Aug2014; remove ~1,200 contaminant assemblies (human,mouse,bacteria,..)	
pubset6	26886	no-omcl 20Aug2014; intron-miss loci, paralog/alt reclass, v2	
pubset7	27775	arp7bor2b orthology completion, 21Aug2014,	
pubset8	28400	arp7bor3b orthology, 21Sep2014, various checks, ~600 missed loci from analyses	
pubset9a	29074	arp7bor4 orthology, 24Sep2014,	
pubset9b	29127	arp7bor5 orthology, 30Sep2014, found 55 ortho-misses	
The EvidentialGene pipeline with associated sources, processing steps and gene set versions is described. The number of input transcripts (Input_Tr), the number retained after each step (NR_out), the *D. magna* gene set associated with each step and the data source (either this study or available at the time of the analysis is shown). The Stages 1–3 refer to the pipeline description in the methods section.			

**Table 2:** RNA—Seq metrics overview.

	**X**	**I**	**XI**
Number of read pairs	3,403,673,296	2,812,630,218	443,120,153
Median read pairs per sample	66,965,781	57,197,308	22,617,799.5
Mean read pairs per sample	68,073,465.92	58,596,462.88	24,617,786.28
Number of reads pairs with phred score >30	2,621,703,528	2,273,042,316	341,213,774
Mean phred score per sequence	32.67	33.29	33.73
Median phred score per sequence	35.00	36.00	35.86
Number of environmental exposures	8	8	6
Number of libraries	25	24	18
The number of total read pairs refers to the total pair read counts per genotype. The median and mean read pairs per sample refer to the sample specific read pairs, where samples constitute the individual exposures including multiple biological replicates of the same genotype per condition. In addition, the fraction of read pairs with phred >30 with respective mean and median values are shown. Number of environmental exposures indicates the number of environmental perturbations to which the genotypes were exposed. The number of libraries constructed per sample is shown; for the X genotype 25 libraries were constructed, including 2 controls as the exposures were completed over two days. For the I genotype 24 libraries were constructed. For the XI genotype 18 libraries were constructed.			

**Table 3:** Summary of RNA-Seq raw data metrics

**Sample ID**	**Genotype**	**Sample Code**	**Treatment Code**	**Replicate**	**Raw Read Pairs**	**Trimmed Read Pairs**	**Percentage**	**Insert_Size**	**StdDev**
I_CO_r1_03	I	Dman_03	CO	r1	63,494,959.000	49,603,877.000	78.12%	289	137
I_CA_r1_05	I	Dman_05	CA	r1	62,993,811.000	49,028,587.000	77.83%	311	188
I_BX_r1_09	I	Dman_09	BX	r1	60,794,872.000	48,712,777.000	80.13%	292	156
I_TR_r1_13	I	Dman_13	TR	r1	55,021,109.000	43,773,368.000	79.56%	319	173
I_FI_r1_15	I	Dman_15	FI	r1	58,768,662.000	48,858,401.000	83.14%	276	143
I_BN_r1_21	I	Dman_21	BN	r1	68,881,858.000	55,980,417.000	81.27%	272	116
I_PA_r1_23	I	Dman_23	PA	r1	73,454,356.000	60,869,142.000	82.87%	298	141
I_CR_r1_27	I	Dman_27	CR	r1	64,360,226.000	53,858,355.000	83.68%	287	90
I_CO_r2_29	I	Dman_29	CO	r2	53,507,535.000	44,845,409.000	83.81%	268	144
I_CO_r3_31	I	Dman_31	CO	r3	50,103,302.000	44,154,666.000	88.13%	277	151
I_CA_r2_35	I	Dman_35	CA	r2	54,459,613.000	47,952,269.000	88.05%	262	92
I_BX_r2_36	I	Dman_36	BX	r2	58,852,394.000	51,366,050.000	87.28%	289	139
I_BX_r3_38	I	Dman_38	BX	r3	48,433,025.000	38,663,288.000	79.83%	265	87
I_FI_r2_39	I	Dman_39	FI	r2	51,705,574.000	41,476,171.000	80.22%	269	153
I_FI_r3_41	I	Dman_41	FI	r3	48,109,963.000	38,197,802.000	79.40%	271	146
I_CA_r3_44	I	Dman_44	CA	r3	56,372,343.000	45,134,149.000	80.06%	255	89
I_BN_r2_46	I	Dman_46	BN	r2	54,184,465.000	44,125,200.000	81.44%	298	130
I_BN_r3_47	I	Dman_47	BN	r3	47,781,030.000	39,153,548.000	81.94%	304	195
I_CR_r2_50	I	Dman_50	CR	r2	48,214,069.000	40,159,238.000	83.29%	279	120
I_CR_r3_52	I	Dman_52	CR	r3	51,730,328.000	42,678,747.000	82.50%	283	121
I_TR_r2_53	I	Dman_53	TR	r2	58,022,273.000	46,414,803.000	79.99%	283	150
I_TR_r3_54	I	Dman_54	TR	r3	82,196,854.000	70,756,025.000	86.08%	295	120
I_PA_r2_56	I	Dman_56	PA	r2	72,623,134.000	62,031,907.000	85.42%	288	122
I_PA_r3_58	I	Dman_58	PA	r3	62,249,354.000	51,270,680.000	82.36%	292	88
X_CO_r1_32	X	Dman_32	CO	r1	71,868,056.000	54,279,251.000	75.53%	278	111
X_CA_r1_33	X	Dman_33	CA	r1	52,241,312.000	39,732,399.000	76.06%	286	152
X_FI_r1_42	X	Dman_42	FI	r1	71,287,854.000	57,746,708.000	81.00%	283	172
X_BN_r1_45	X	Dman_45	BN	r1	61,527,322.000	49,445,396.000	80.36%	280	132
X_BX_r1_48	X	Dman_48	BX	r1	66,965,781.000	50,685,192.000	75.69%	266	114
X_CR_r1_51	X	Dman_51	CR	r1	76,818,434.000	59,552,351.000	77.52%	246	87
X_PA_r1_55	X	Dman_55	PA	r1	58,307,384.000	44,248,248.000	75.89%	251	85
X_CA_r2_60	X	Dman_60	CA	r2	65,880,062.000	50,044,254.000	75.96%	237	81
X_CA_r3_61	X	Dman_61	CA	r3	66,867,949.000	50,405,221.000	75.38%	232	83
X_CO_r2_62	X	Dman_62	CO	r2	67,659,124.000	51,393,424.000	75.96%	241	125
X_FI_r2_64	X	Dman_64	FI	r2	70,280,087.000	54,395,752.000	77.40%	243	85
X_FI_r3_65	X	Dman_65	FI	r3	66,226,108.000	50,516,556.000	76.28%	255	116
X_BX_r2_67	X	Dman_67	BX	r2	67,379,171.000	51,252,578.000	76.07%	286	145
X_BX_r3_68	X	Dman_68	BX	r3	81,455,682.000	67,969,702.000	83.44%	291	111
X_TR_r1_70	X	Dman_70	TR	r1	57,871,883.000	48,655,208.000	84.07%	279	163
X_TR_r2_73	X	Dman_73	TR	r2	58,433,654.000	49,868,737.000	85.34%	281	144
X_BN_r2_74	X	Dman_74	BN	r2	75,608,849.000	57,220,416.000	75.68%	287	134
X_PA_r2_75	X	Dman_75	PA	r2	63,636,165.000	51,803,268.000	81.41%	261	104
X_CO_r3_76	X	Dman_76	CO	r3	90,321,814.000	73,595,073.000	81.48%	271	101
X_CO_r4_77	X	Dman_77	CO	r4	118,207,131.000	87,998,612.000	74.44%	278	154
X_CR_r2_78	X	Dman_78	CR	r2	62,101,323.000	54,966,131.000	88.51%	287	139
X_CR_r3_79	X	Dman_79	CR	r3	90,997,555.000	78,494,531.000	86.26%	254	160
X_PA_r3_80	X	Dman_80	PA	r3	58,790,865.000	50,985,812.000	86.72%	43	112
X_BN_r3_83	X	Dman_83	BN	r3	83,174,861.000	71,186,306.000	85.59%	228	141
X_TR_r3_84	X	Dman_84	TR	r3	46,523,201.000	33,845,684.000	72.75%	226	117
XI_CD_r1_01	XI	XI_01	CD	r1	35,429,813.000	35,359,972.000	99.80%	166	53
XI_CD_r2_02	XI	XI_02	CD	r2	58,550,652.000	58,459,895.000	99.84%	166	54
XI_CD_r3_03	XI	XI_03	CD	r3	16,981,329.000	16,957,777.000	99.86%	164	53
XI_CO_r1_04	XI	XI_04	CO	r1	17,549,155.000	17,526,674.000	99.87%	173	98
XI_CO_r2_05	XI	XI_05	CO	r2	12,968,443.000	12,951,973.000	99.87%	161	52
XI_CO_r3_06	XI	XI_06	CO	r3	22,191,696.000	22,165,545.000	99.88%	166	150
XI_PB_r1_07	XI	XI_07	PB	r1	13,209,418.000	13,022,646.000	98.59%	160	49
XI_PB_r2_08	XI	XI_08	PB	r2	25,098,583.000	25,063,912.000	99.86%	168	121
XI_PB_r3_09	XI	XI_09	PB	r3	20,147,612.000	20,119,479.000	99.86%	164	53
XI_PH5_r1_10	XI	XI_10	PH5	r1	17,117,432.000	16,780,537.000	98.03%	160	48
XI_PH5_r2_11	XI	XI_11	PH5	r2	23,043,903.000	22,676,257.000	98.40%	158	49
XI_PH5_r3_12	XI	XI_12	PH5	r3	28,299,589.000	27,784,289.000	98.18%	163	51
XI_NaCl_r1_13	XI	XI_13	NaCl	r1	9,454,837.000	9,279,727.000	98.15%	167	50
XI_NaCl_r2_14	XI	XI_14	NaCl	r2	14,129,350.000	13,855,321.000	98.06%	165	49
XI_NaCl_r3_15	XI	XI_15	NaCl	r3	36,387,177.000	35,659,077.000	98.00%	164	50
XI_UV_r1_16	XI	XI_16	UV	r1	35,314,952.000	34,645,324.000	98.10%	166	49
XI_UV_r2_17	XI	XI_17	UV	r2	33,396,591.000	32,775,509.000	98.14%	167	49
XI_UV_r3_18	XI	XI_18	UV	r3	23,849,621.000	23,398,424.000	98.11%	166	49
Read pair counts are shown per individual sample. The percentage of retained reads after trimming as well as insert size (st dev) per RNA sample and replica are shown. The sample ID consists of the **genotype name** -X, I or XI-; **the environmental perturbation**—FI: fish; TR: *Triops*; PA: *P ramosa* parasite; BX: toxic cyanobacteria strain; BN: non-toxic cyanobacteria strain; CR: crowding; CA: carbaryl; CD: cadmium; PB: lead; PH5; UV: UV exposure; NaCl: Sodium Chloride; CO: control-; **replica-sample code for sequence submission**. The protocols run on the three strains, their biological replicates and the associated NCBI Sequence Read Archive run accession numbers are listed in Supplementary Table 2.									

**Table 4:** Mapping metrics

**Sample ID**	**Input**	**Mapped**	**Percentage**	**Unique**	**PercentU**	**Multiple**	**PercentM**
I_BN_r1_21	111,960,834	80,083,757	71.53%	66,413,218	82.93%	13,670,539	17.07%
I_BN_r2_46	88,250,400	65,226,041	73.91%	52,033,898	79.77%	13,192,143	20.23%
I_BN_r3_47	78,307,096	59,500,967	75.98%	48,036,297	80.73%	11,464,670	19.27%
I_BX_r1_09	97,425,554	74,283,576	76.25%	62,543,895	84.20%	11,739,681	15.80%
I_BX_r2_36	102,732,100	77,145,075	75.09%	61,447,456	79.65%	15,697,619	20.35%
I_BX_r3_38	77,326,576	58,031,769	75.05%	46,638,628	80.37%	11,393,141	19.63%
I_CA_r1_05	98,057,174	75,699,788	77.20%	64,032,567	84.59%	11,667,221	15.41%
I_CA_r2_35	95,904,538	71,544,033	74.60%	57,839,196	80.84%	13,704,837	19.16%
I_CA_r3_44	90,268,298	64,558,279	71.52%	52,145,719	80.77%	12,412,560	19.23%
I_CO_r1_03	99,207,754	74,962,960	75.56%	63,416,691	84.60%	11,546,269	15.40%
I_CO_r2_29	89,690,818	65,887,322	73.46%	53,024,248	80.48%	12,863,074	19.52%
I_CO_r3_31	88,309,332	66,542,581	75.35%	54,249,318	81.53%	12,293,263	18.47%
I_CR_r1_27	107,716,710	65,832,256	61.12%	55,207,910	83.86%	10,624,346	16.14%
I_CR_r2_50	80,318,476	60,003,726	74.71%	48,426,482	80.71%	11,577,244	19.29%
I_CR_r3_52	85,357,494	63,331,741	74.20%	51,823,192	81.83%	11,508,549	18.17%
I_FI_r1_15	97,716,802	69,325,492	70.95%	57,272,131	82.61%	12,053,361	17.39%
I_FI_r2_39	82,952,342	60,811,958	73.31%	49,298,557	81.07%	11,513,401	18.93%
I_FI_r3_41	76,395,604	57,925,361	75.82%	46,653,689	80.54%	11,271,672	19.46%
I_PA_r1_23	121,738,284	85,390,378	70.14%	71,455,076	83.68%	13,935,302	16.32%
I_PA_r2_56	124,063,814	92,077,396	74.22%	75,330,778	81.81%	16,746,618	18.19%
I_PA_r3_58	102,541,360	76,400,295	74.51%	61,663,768	80.71%	14,736,527	19.29%
I_TR_r1_13	87,546,736	64,947,049	74.19%	55,400,302	85.30%	9,546,747	14.70%
I_TR_r2_53	92,829,606	66,809,655	71.97%	54,544,356	81.64%	12,265,299	18.36%
I_TR_r3_54	141,512,050	104,106,636	73.57%	84,382,620	81.05%	19,724,016	18.95%
X_BN_r1_45	98,890,792	71,840,880	72.65%	59,566,099	82.91%	12,274,781	17.09%
X_BN_r2_74	114,440,832	85,650,437	74.84%	71,414,344	83.38%	14,236,093	16.62%
X_BN_r3_83	142,372,612	108,233,621	76.02%	86,494,740	79.91%	21,738,881	20.09%
X_BX_r1_48	101,370,384	71,969,721	71.00%	59,654,388	82.89%	12,315,333	17.11%
X_BX_r2_67	102,505,156	77,480,991	75.59%	64,605,122	83.38%	12,875,869	16.62%
X_BX_r3_68	135,939,404	100,320,333	73.80%	83,979,132	83.71%	16,341,201	16.29%
X_CA_r1_33	79,464,798	55,469,313	69.80%	46,582,761	83.98%	8,886,552	16.02%
X_CA_r2_60	100,088,508	75,112,653	75.05%	60,181,010	80.12%	14,931,643	19.88%
X_CA_r3_61	100,810,442	74,154,574	73.56%	60,414,064	81.47%	13,740,510	18.53%
X_CO_r1_32	108,558,502	81,543,420	75.11%	67,655,636	82.97%	13,887,784	17.03%
X_CO_r2_62	102,786,848	75,848,649	73.79%	62,061,875	81.82%	13,786,774	18.18%
X_CO_r3_76	147,190,146	106,434,191	72.31%	88,808,043	83.44%	17,626,148	16.56%
X_CO_r4_77	175,997,224	122,205,443	69.44%	101,558,190	83.10%	20,647,253	16.90%
X_CR_r1_51	119,104,702	81,268,612	68.23%	67,293,396	82.80%	13,975,216	17.20%
X_CR_r2_78	109,932,262	79,815,181	72.60%	66,586,543	83.43%	13,228,638	16.57%
X_CR_r3_79	156,989,062	99,288,707	63.25%	82,718,053	83.31%	16,570,654	16.69%
X_FI_r1_42	115,493,416	84,630,965	73.28%	70,317,972	83.09%	14,312,993	16.91%
X_FI_r2_64	108,791,504	79,180,393	72.78%	65,196,666	82.34%	13,983,727	17.66%
X_FI_r3_65	101,033,112	74,878,527	74.11%	60,869,160	81.29%	14,009,367	18.71%
X_PA_r1_55	88,496,496	65,061,089	73.52%	52,747,415	81.07%	12,313,674	18.93%
X_PA_r2_75	103,606,536	75,988,380	73.34%	62,713,686	82.53%	13,274,694	17.47%
X_PA_r3_80	101,971,624	76,658,837	75.18%	62,960,881	82.13%	13,697,956	17.87%
X_TR_r1_70	97,310,416	72,713,118	74.72%	60,631,515	83.38%	12,081,603	16.62%
X_TR_r2_73	99,737,474	70,684,693	70.87%	59,520,497	84.21%	11,164,196	15.79%
X_TR_r3_84	67,691,368	49,660,414	73.36%	40,536,759	81.63%	9,123,655	18.37%
XI_CD_r1_01	70,719,944	54,379,411	76.89%	42,974,691	79.03%	11,404,720	20.97%
XI_CD_r2_02	116,919,790	90,081,361	77.05%	71,488,938	79.36%	18,592,423	20.64%
XI_CD_r3_03	33,915,554	25,034,185	73.81%	19,821,595	79.18%	5,212,590	20.82%
XI_CO_r1_04	35,053,348	26,605,116	75.90%	21,802,150	81.95%	4,802,966	18.05%
XI_CO_r2_05	25,903,946	19,443,392	75.06%	15,771,894	81.12%	3,671,498	18.88%
XI_CO_r3_06	44,331,090	34,471,382	77.76%	28,113,780	81.56%	6,357,602	18.44%
XI_NaCl_r1_13	18,559,454	13,363,593	72.00%	11,155,291	83.48%	2,208,302	16.52%
XI_NaCl_r2_14	27,710,642	20,737,809	74.84%	17,426,679	84.03%	3,311,130	15.97%
XI_NaCl_r3_15	71,318,154	52,907,126	74.18%	44,197,531	83.54%	8,709,595	16.46%
XI_PH5_r1_10	33,561,074	25,250,026	75.24%	20,629,525	81.70%	4,620,501	18.30%
XI_PH5_r2_11	45,352,514	33,802,651	74.53%	27,780,026	82.18%	6,022,625	17.82%
XI_PH5_r3_12	55,568,578	41,739,579	75.11%	34,148,330	81.81%	7,591,249	18.19%
XI_PB_r1_07	26,045,292	19,574,223	75.15%	15,859,586	81.02%	3,714,637	18.98%
XI_PB_r2_08	50,127,824	38,936,820	77.68%	31,320,244	80.44%	7,616,576	19.56%
XI_PB_r3_09	40,238,958	30,335,619	75.39%	24,569,127	80.99%	5,766,492	19.01%
XI_UV_r1_16	69,290,648	51,584,530	74.45%	43,040,794	83.44%	8,543,736	16.56%
XI_UV_r2_17	65,551,018	48,492,486	73.98%	40,789,403	84.11%	7,703,083	15.89%
XI_UV_r3_18	46,796,848	35,412,890	75.67%	30,005,050	84.73%	5,407,840	15.27%
Absolute number and percentage of reads mapped onto the hybrid transcriptome assembly obtained using EvidentialGene. Input: number of raw reads; mapped: number of reads mapping onto the reference transcriptome; percentage: percentage of reads mapping onto the reference transcriptome; unique: reads mapping to a unique location in the reference transcriptome with corresponding percentage (percentU); multiple: reads mapping to multiple locations in the reference transcriptome with corresponding percentage (percentM). The sample names are as in Table 3.							

**Table 5:** RNA-Seq read mapping statistics.

**Strain**	**mRNA set**	**TotR**	**MapR**	**%Map**
X	all	3233374500	3172301416	98.1%
I	all	2789627581	2736214261	98.1%
XI	all	885996197	857334019	96.8%
X	primary	3233374500	2814739850	87.1%
I	primary	2789627581	2429376789	87.1%
XI	primary	885996197	791867853	89.4%
RNA-Seq reads mapping onto *Daphnia magna* transcripts for the X, I and XI genotypes are shown for alternates (all) and primary transcripts (mRNA set). Read pairs were mapped to transcripts with GSNAP (2014-05-15, opts:-N 0 --gmap-mode=none --pairexpect=400). The total number of reads (TotR), the number of mapper reads (MapR) and the percentage of mapped reads (%Map) is shown.				

**Table 6:** Transcripts statistics.

**Strain**	**X**	**I**	**XI**
Number of Transcripts	27,441	28,187	26,508
Length of Transcripts	48,072,095	48,822,339	47,088,659
Number of Bases Mapped	253,948,429,576	217,093,998,202	91,961,017,164
Coverage (bp)	5,282.66	4,446.61	1,952.93
The number of transcripts retained after trimming, their length, the total number of bases mapped and the total coverage (in bp) per sample are shown.			

**Table 7:** Allelic variants.

**Alleles**	**X**	**I**	**XI**
**≤2**	17,252	23,329	23,436
**3**	607	580	614
**4**	45	25	24
Allelic variants identified in the three genotypes used in this study as compared to the reference set of single copy genes from the *D. magna* consensus transcriptome are shown. A cut-off of 1% was applied before allelic variants call.			

**Table 8:** Gene set completeness.

**Species**	**aaSize**	**Frag%**	**OrMiss**	**OrGroup**
*Daphnia magna*	46	1.8%	18	11523
*Daphnia pulex*	−25	5.1%	36	11670
*Tribolium castaneum*	−26	4.1%	42	8765
*Apis mellifera*	38	3.1%	161	8682
*Drosophila melanogaster*	68	1.8%	203	7801
Completeness of species gene sets is measured with average protein sizes and orthology group presence with OrthoMCL analysis. aaSize: average deviation from reference species protein sizes; Fragment%: percent gene size outliers below 2 s.d. of group median size; OrMiss: number of missed ortho-groups that are common to other species; OrGroup: number of orthology groups found.				
