# *Daphnia magna* transcriptome by RNA-Seq across 12 environmental stressors

**DOI:** 10.1038/sdata.2017.6

**Published:** 2017-01-31

**Authors:** Luisa Orsini, Donald Gilbert, Ram Podicheti, Mieke Jansen, James B. Brown, Omid Shams Solari, Katina I. Spanier, John K. Colbourne, Douglas B. Rusch, Ellen Decaestecker, Jana Asselman, Karel A.C. De Schamphelaere, Dieter Ebert, Christoph R. Haag, Jouni Kvist, Christian Laforsch, Adam Petrusek, Andrew P. Beckerman, Tom J. Little, Anurag Chaturvedi, Michael E. Pfrender, Luc De Meester, Mikko J. Frilander

*Scientific Data* 3:160030 doi:10.1038/sdata.2016.30 (2016); Published 10 May 2016; Updated 31 Jan 2017

The original version of this Data Descriptor contained a typographical error in the spelling of the author Douglas B. Rusch, which was incorrectly given as Douglas Rush. This has now been corrected in the PDF and HTML versions of the Data Descriptor.

